# 磁性双功能材料的制备及用于血清中外泌体的捕获和磷肽富集

**DOI:** 10.3724/SP.J.1123.2025.04034

**Published:** 2026-03-08

**Authors:** Zirui WANG, Haijiao ZHENG, Qiong JIA

**Affiliations:** 吉林大学化学学院，吉林 长春 130012; College of Chemistry，Jilin University，Changchun 130012

**Keywords:** 血清外泌体, 钛离子, 磁性固相萃取, 磷肽, 质谱检测, serum exosomes, titanium ion, magnetic solid-phase extraction, phosphopeptides, mass spectrometric detection

## Abstract

作为临床样本，血清因其易于获取等优势被广泛应用于疾病研究。血清中的外泌体不仅携带多种蛋白质、核酸等生物活性物质，还可以参与细胞之间的通讯，与多种疾病的发生发展相关联。作为关键的翻译后修饰之一，蛋白质磷酸化通过动态调控细胞信号网络参与疾病发生发展，其在外泌体中的特异性表达为疾病诊断提供了新视角。本论文设计制备了一种基于2-三氟甲基-4-氨基苯甲酸和Ti^4+^的磁性双功能材料（MagphTi^4+^），通过2-三氟甲基-4-氨基苯甲酸的疏水作用和Ti^4+^的静电作用共同锚定外泌体的双层膜，通过基质的磁响应性质实现外泌体的分离提取。同时，利用材料表面的Ti^4+^和磷酸根的螯合作用实现磷肽的富集。通过冷场发射扫描电子显微镜、热重分析、红外光谱、X射线光电子能谱和Zeta电位测定对MagphTi^4+^进行了表征。通过冷场发射扫描电子显微镜、透射电子显微镜和十二烷基硫酸钠聚丙烯酰胺凝胶电泳对外泌体进行了表征，验证了MagphTi^4+^对外泌体精准识别和快速分离的能力。以*β*-酪蛋白酶解物为标准磷肽模型，建立了磁性固相萃取（MSPE）-质谱检测技术平台，实现了MagphTi^4+^对磷肽的高选择性（*β*-酪蛋白与牛血清白蛋白的质量比为1∶5 000）和高灵敏度的检测（检出限低至0.4 fmol/μL）。将该方法用于实际样品分析，实现了对血清外泌体的提取与低丰度磷肽的分离富集。本方法有望用于临床血清样本中外泌体的高通量磷酸化蛋白质组学分析和疾病标志物的筛查。

## Abstract (en)

As a clinical sample， serum is widely used in disease research due to the minimally invasive nature of its collection and easy accessibility. Exosomes in serum not only carry various bioactive substances such as proteins and nucleic acids but also participate in intercellular communication， which is closely associated with the development of multiple diseases. As one of the most critical post-translational modifications， protein phosphorylation dynamically regulates cellular signaling networks and participates in disease pathogenesis. However， due to the low abundance of exosomes in serum and the fact that phosphoproteins constitute only 1%–2% of the total proteome， direct analysis of exosomal phosphoproteins from serum faces significant challenges. The specific expression of phosphoproteins in exosomes provides novel perspectives for disease diagnosis. In this study， magnetic bifunctional nanoparticles （MagphTi^4+^） based on 2-trifluoromethyl-4-aminobenzoic acid and Ti^4+^ were synthesized， which anchor the exosome bilayer membrane through the hydrophobic interactions of 2-trifluoromethyl-4-aminobenzoic acid and the electrostatic interactions of Ti^4+^， and the magnetic responsiveness ensures the isolation of exosomes. Simultaneously， the enrichment of phosphopeptides was achieved by the chelation between Ti^4+^ and phosphate groups. MagphTi^4+^ was characterized by cold field scanning electron microscopy， thermogravimetric analysis， Fourier transform infrared spectroscopy， X-ray photoelectron spectroscopy and zeta potential measurement， demonstrating the successful synthesis and functional characteristics of MagphTi^4+^. The precise recognition and rapid separation of exosomes were validated by cold field scanning electron microscopy， transmission electron microscopy and sodium dodecyl sulfate-polyacrylamide gel electrophoresis. Using *β*-casein as a standard phosphoprotein model， a magnetic solid-phase extraction （MSPE）-matrix-assisted laser desorption ionization-time of flight mass spectrometry platform was established， which demonstrated high selectivity （mass ratio of *β*-casein to bovine serum albumin was 1∶5 000） and sensitivity （detection limit was 0.4 fmol/μL） for phosphopeptide detection. Subsequently， the method was applied to saliva and nonfat milk samples； 11 phosphopeptides were identified in saliva and 20 were identified in nonfat milk， which demonstrates excellent enrichment capability for phosphopeptide detection in real samples. When applied to clinical serum samples， the method successfully achieved the extraction of exosomes in serum and enrichment of phosphopeptides with low abundance. This integrated approach provides a powerful tool for exosome capture and phosphopeptide enrichment from complex biological samples， showing significant potential for high-throughput phosphoproteomic analysis of exosomes from clinical serum and the screening of disease biomarkers. Further application of the method developed in this study holds promise for elucidating the mechanisms of exosome-mediated intercellular transmission of phosphorylation signaling， thereby identifying novel targets related to tumor metastasis and immune escape.

血液蛋白质组学在后基因组时代的生物医学研究中起着至关重要的作用，在生物标志物研究领域备受关注^［1，2］^。研究人员通过高通量的蛋白质组学检测技术，可以分析血清样品中的多种蛋白质成分，包括总蛋白质组及蛋白质的翻译后修饰，为新型生物标志物的发现和临床诊断方法的开发提供帮助^［3-5］^。外泌体是一种运载大量功能性蛋白质的微小囊泡，在体内参与信号转导、肿瘤发生、免疫反应、细胞通讯等生理过程，和多种疾病的发生与进展存在密切关联^［6-8］^。近年来，血清外泌体蛋白质组学的研究已深入肿瘤微环境调控、疾病标志物筛选及药物靶向递送系统开发等前沿方向^［9-11］^，成为临床样品分析的热点。作为关键的翻译后修饰之一，蛋白质磷酸化可通过精确调控信号转导、代谢重编程等过程参与调节几乎所有必要的细胞功能，可动态反映机体的病理状态，在疾病的早期诊断与疗效监测中具有显著潜力^［12-14］^。因此，血清外泌体的磷酸化蛋白质组学研究具有重要的临床意义。

随着色谱-质谱联用技术的发展，该技术已成为研究外泌体蛋白质组学的强有力工具之一。然而，由于血清中外泌体含量和发生磷酸化修饰的蛋白质含量（仅占蛋白质总量的1%~2%）均极低，因此难以实现未经前处理步骤的样品的直接检测。为了解决这一问题，一些复杂样品中的外泌体提取方法和蛋白质磷酸化富集手段应运而生^［15-17］^。传统的外泌体分离方法主要有尺寸排阻法、超速离心法、免疫亲和法。这些方法虽然可以实现外泌体的提取，但仍存在一些问题，如超速离心法耗时长且回收率不足30%^［18］^；免疫亲和法成本高、洗脱效率低等^［19］^。结合磁性分离介质和特异性识别基团选择性捕获外泌体是一种有效的策略^［20-22］^，而开发新型外泌体磁分离技术是提升检测准确度的突破口。由于外泌体具有疏水的磷脂双分子层且膜表面带有负电荷，近年来，开发带正电的磁性疏水材料对外泌体进行捕获已成为研究热点^［23-25］^。

目前，蛋白质磷酸化的富集手段主要包括金属氧化物亲和色谱法（MOAC）、离子交换色谱法（IEX）、固定化金属离子亲和色谱法（IMAC）^［26-28］^。其中，IMAC技术由于具有简单、实用等优点，已成为磷肽富集最重要的方法^［29］^。其富集机理为在酸性条件下通过金属离子与磷酸基团的螯合作用捕获磷肽，而捕获的磷肽可以在碱性条件下被洗脱^［30］^。近年来，一些研究者将IMAC技术用于外泌体磷酸化蛋白质组的分析，取得了令人满意的结果。例如，2023年，Tao等^［31］^将Ti^4+^与八聚精氨酸共修饰于磁珠表面，开发出用于体液中细胞外囊泡分离与细胞外囊泡中磷肽原位富集（EVTOP）的一体化平台，实现了尿液、血清、脑脊液等多种复杂生物流体中外泌体捕获、裂解、酶解与磷肽富集的全流程集成，最大程度上减小了样品损失，从100 μL脑脊液中鉴定出1 249种磷肽。2024年，Fang等^［32］^将Ti^4+^与核酸修饰在磁珠表面，开发的外泌体高效分离平台可在5 min内以92%的回收率实现对模型外泌体的高效捕获，并从100 μL血清样品中鉴定出了320种外泌体蛋白质。

针对外泌体膜的疏水性、负电性与磷肽的负电性，本研究设计了一种新型基于Ti^4+^的疏水性磁性纳米颗粒（MagphTi^4+^），通过2-三氟甲基-4-氨基苯甲酸（CF_3_C_6_H_3_-2-（NH_2_）CO_2_H）的疏水作用和Ti^4+^的静电作用实现了外泌体的捕获，通过IMAC实现了磷肽的富集。首先，通过硅酸四乙酯（TEOS）和3-氨丙基三乙氧基硅烷（APTES）对Fe_3_O_4_进行硅烷化，得到氨基修饰的核壳型磁性基质。其次，通过1-（3-二甲氨基丙基）-3-乙基碳二亚胺盐酸盐（EDC）/*N*-羟基丁二酰亚胺（NHS）活化过程将2-三氟甲基-4-氨基苯甲酸偶联到基质表面；最后，通过Ti^4+^与三氟甲基分子间的作用将Ti^4+^螯合到材料表面。利用MagphTi^4+^双功能磁性材料的疏水性和正电性实现复杂样品中外泌体的特异性捕获和快速分离。同时，利用MagphTi^4+^表面的Ti^4+^实现对磷肽的特异性富集。以标准磷酸化蛋白酶解所得到的磷肽评价方法的灵敏度和选择性，以牛奶和唾液样品评价方法在复杂样品分析中对磷肽的富集能力。最后，将此方法用于血清外泌体的分离和外泌体磷肽的富集分析中，综合考察了本方法在实际样品分析中的适用性。

## 1 实验部分

### 1.1 仪器、试剂与材料

SU8020型冷场发射扫描电镜（SEM）、HT7820型透射电子显微镜（TEM）（Hitachi，Japan）；Q500型热重分析仪（TGA，TA，USA）；SQNicolet IS5型傅里叶变换红外光谱仪（FTIR，Bruker，Germany）；ESCALAB250型X射线光电子能谱仪（XPS，Thermo，USA）；Zetasizer Nano ZS ZEN3600型ZETA 电位和纳米粒度分析仪（Zeta，Malvern，Britain）；JY-SCZ2型电泳仪（SDS-PAGE，北京君意，中国）；AB Sciex 5800型基质辅助激光解析电离-飞行时间质谱仪（MALDI-TOF MS，AB SCIEX，USA）。

TEOS（纯度≥98%）、2-三氟甲基-4-氨基苯甲酸（纯度≥98%）、硫酸氧钛-硫酸水合物（TiOSO_4_·*x*H_2_SO_4_·*x*H_2_O，纯度≥93%）、考马斯亮蓝（纯度≥70%）、乙腈（纯度99%，ACN）、3-氨丙基三乙氧基硅烷（纯度≥98%）、*N*-羟基丁二酰亚胺（纯度≥98%）、2，5-二羟基苯甲酸（DHB，纯度99%）、尿素（H_2_NCONH_2_，纯度99%）、硫脲（CH_4_N_2_S，纯度99%）、二硫苏糖醇（DTT，纯度≥98%）、碘乙酰胺（IAA，纯度≥98%）、三氟乙酸（TFA，纯度99%）、苯甲基磺酰氟（PMSF，纯度≥98%）、甲酸（FA，纯度≥98%）、1-（3-二甲氨基丙基）-3-乙基碳二亚胺盐酸盐（纯度≥98%）PBS溶液（pH 7.2~7.4）、磷酸（纯度85%）购于Aladdin试剂（上海）公司；胰蛋白酶（trypsin，纯度≥98%）、牛血清白蛋白（BSA，纯度≥98%）、*β*-酪蛋白（*β*-casein，纯度99%）购于Sigma试剂有限公司；三氯化铁（FeCl_3_·6H_2_O，纯度99%）、醋酸钠（NaAc，纯度≥98%）、盐酸（HCl，纯度35%）、氨水（NH_3_·H_2_O，纯度25%）、无水乙醇（EtOH，纯度99%）、乙二醇（EG，纯度99%）、三乙胺（纯度99.5%）、磷钨酸水合物（H_3_O_40_PW_12_·*x*H_2_O，纯度≥99.995%）、氯化钠（NaCl，纯度99%）、碳酸氢铵（NH_4_HCO_3_，纯度99%）、三氯甲烷（CHCl_3_，纯度99%）购于北京化学试剂公司。脱脂牛奶样品购于长春本地超市；人血清样本和唾液样本来源于吉林大学中日联谊医院，研究经吉林大学中日联谊医院伦理委员会批准，批准号为2021113000。

### 1.2 实验方法

#### 1.2.1 MagphTi^4+^的合成

称取2.6 g FeCl_3_·6H_2_O和5.9 g NaAc溶于90 mL EG中，将溶液转移至反应釜中，于200 ℃加热15 h。产物用乙醇和纯水交替冲洗6次，磁分离得到Fe_3_O_4_磁性内核^［33］^。取400 mg Fe_3_O_4_置于含76 mL纯水、290 mL无水乙醇和2.1 mL氨水的混合溶液中，以300 W功率超声15 min使磁球分散均匀，加入1.9 mL TEOS，于40 ℃、50 r/min的条件下搅拌15 h，磁分离得到硅烷化的磁球（简称Fe_3_O_4_-SiO_2_）。

取90 mg Fe_3_O_4_-SiO_2_置于22 mL乙醇中，以300 W功率超声15 min使其分散均匀，加入0.65 mL APTES，于65 ℃、50 r/min的条件下搅拌20 h，经磁吸分离得到氨基修饰的磁球（简称Fe_3_O_4_-NH_2_）^［34］^。

称取370 mg 2-三氟甲基-4-氨基苯甲酸，3.0 g EDC和2.4 g NHS，溶于28 mL纯水，混合均匀后加入Fe_3_O_4_-NH_2_，于37 ℃、50 r/min的条件下搅拌24 h，经磁吸分离得到2-三氟甲基-4-氨基苯甲酸修饰的磁性微球（简称Magph）。

取370 mg Magph分散到30 mL纯水中，加入1.3 g硫酸氧钛-硫酸水合物与1 μL 浓盐酸（12 mol/L），于37 ℃、50 r/min的条件下搅拌20 h。反复用乙醇和纯水交替清洗产物，经磁分离得到MagphTi^4+［35］^。

#### 1.2.2 样品的前处理

在之前工作^［36，37］^的基础上对实际生物样品处理过程进行改进。在150 μL脱脂牛奶样品中加入300 μL含有40 mmol/L NH_4_HCO_3_的缓冲溶液，于4 000 r/min、4 ℃的条件下离心16 min，上清液于95 ℃油浴中加热15 min使蛋白变性，降至室温后加入15 μg胰蛋白酶，于37 ℃孵育14 h，将制备的肽段溶液置于冰箱中冷冻备用。

在100 μL唾液中加入400 μL 40 mmol/L NH_4_HCO_3_的缓冲溶液，于4 000 r/min、4 ℃的条件下离心16 min，取上清液置于冰箱中冷冻备用。

在100 μL血清中加入400 μL 40 mmol/L NH_4_HCO_3_的缓冲溶液，于4 000 r/min、4 ℃的条件下离心16 min。取100 µL上清液，加入到分散有1 mg MagphTi^4+^的100 µL PBS缓冲液（pH=7.4）中，于室温孵育30 min后，磁吸30 s并除去上清液。用100 µL PBS缓冲液（pH=7.4）洗涤磁球，重复3次。将磁球重悬于100 µL含有2 mol/L硫脲、8 mol/L尿素、1 mmol/L PMSF、60 mmol/L DTT的裂解缓冲溶液中，于95 ℃油浴中加热10 min，冷却至室温，加入10 μg胰蛋白酶，于37 ℃孵育14 h，之后置于冰箱中冷冻备用，以备后续分析。

外泌体TEM表征制样：将富集血清外泌体的磁球重悬于100 μL 50 mmol/L的三乙胺溶液中，于常温摇晃30 min后，磁吸30 s并收集上清液。取5 μL上清液与5 μL 2%磷钨酸溶液混合，常温孵育10 min，置于铜网，于常温下干燥。

#### 1.2.3 磷肽的富集

取6.5 mg MagphTi^4+^分散于0.13 mL含1% TFA的70%乙腈水溶液中用作磁球分散液。将冷冻备用的样品于常温下融化，取10 µL待分析的样品溶液和10 µL磁球分散液，加入80 µL含1% TFA的70%乙腈水溶液，超声5 min使磁球均匀分散，常温振荡35 min后，磁吸30 s后分离磁球，用100 µL含1% TFA的70%乙腈水溶液清洗磁球，清洗过程重复3次。最后将磁球分散于25 µL 1%氨水中，常温振荡35 min后，经磁分离收集洗脱液。

#### 1.2.4 MALDI-TOF MS条件

选用反射正离子模式，离子源的温度为110 ℃，电喷雾的电压为2.3 kV，扫描范围为*m/z* 1 000~3 500。取40 mg DHB溶于40 mL 含1% H_3_PO_4_的70%乙腈水溶液充当基质溶液。将等体积基质溶液和样品溶液均匀混合，取1 µL混合均匀的溶液滴于MALDI靶板。

## 2 结果与讨论

### 2.1 MagphTi^4+^的合成与表征

图1为MagphTi^4+^的合成路线图与富集磷肽的流程图。SEM图像展示了Fe_3_O_4_和MagphTi^4+^的形貌和尺寸。如图2a所示，Fe_3_O_4_形貌呈光滑的球体，其直径约为267 nm；如图2b所示，修饰后的磁性颗粒表面粗糙，颗粒直径尺寸略大于Fe_3_O_4_，其直径约为290 nm。

**图1:**
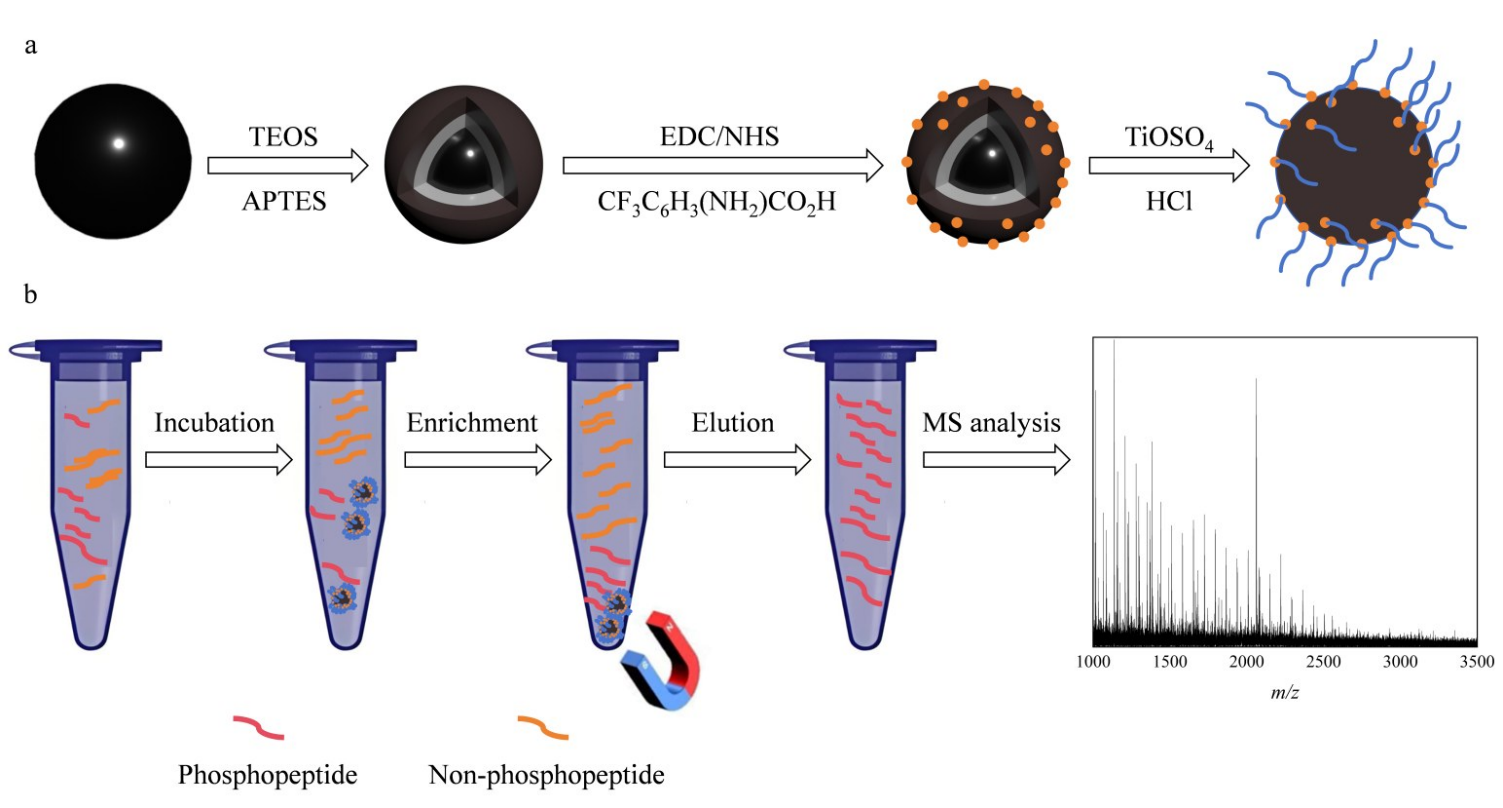
MagphTi^4+^的（a）合成路线及其（b）富集磷肽的过程

**图2:**
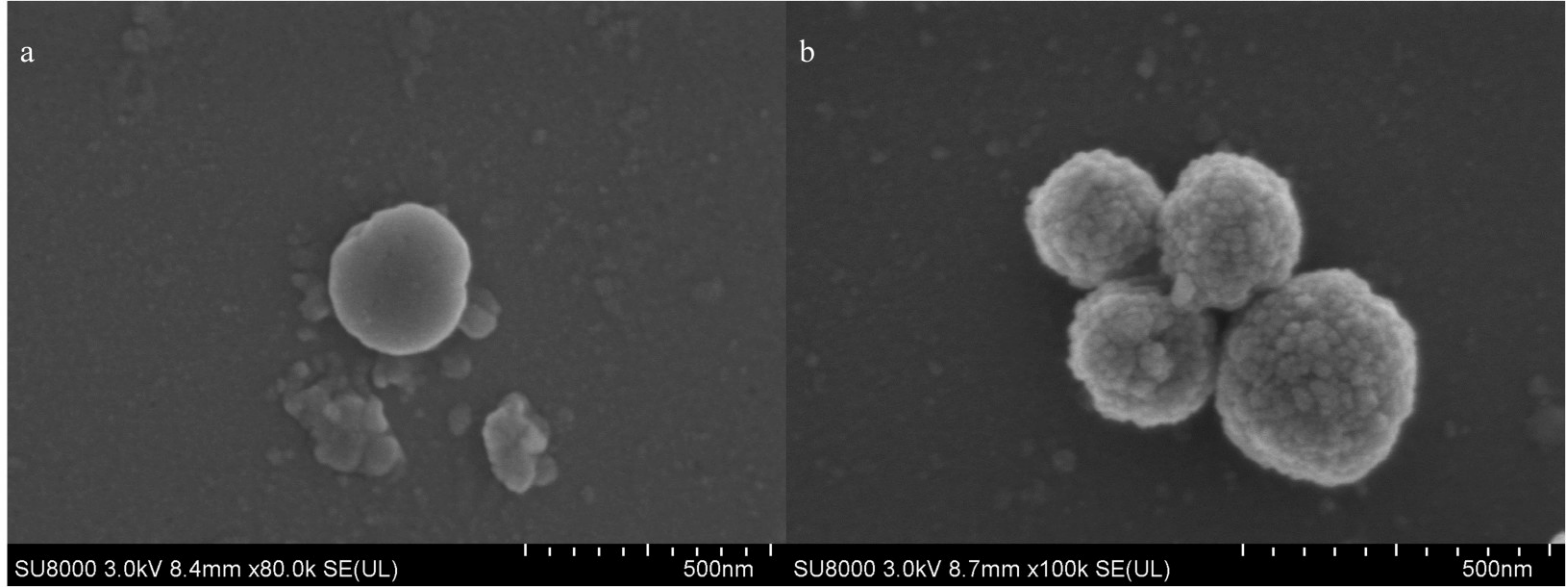
（a）Fe_3_O_4_和（b）MagphTi⁴⁺的SEM图像

采用FTIR对Fe_3_O_4_、Fe_3_O_4_-NH_2_、MagphTi^4+^进行表征。由图3a可见，Fe_3_O_4_、Fe_3_O_4_-NH_2_、MagphTi^4+^在580和1 080 cm^-1^处存在特征吸收峰，可归属于O-Fe键的振动。Fe_3_O_4_-NH_2_曲线中，在1 560 cm^-1^和1 261 cm^-1^处有归属于N-H和C-N的吸收峰，证明APTES成功修饰在磁球表面。MagphTi^4+^光谱中，1 123 cm^-1^处有归属于C-F-C的吸收峰、在3 399 cm^-1^处有归属于酰胺键的特征吸收峰、在697 cm^-1^处有归属于Ti-F-Ti的特征吸收峰，说明MagphTi^4+^制备成功。

**图3:**
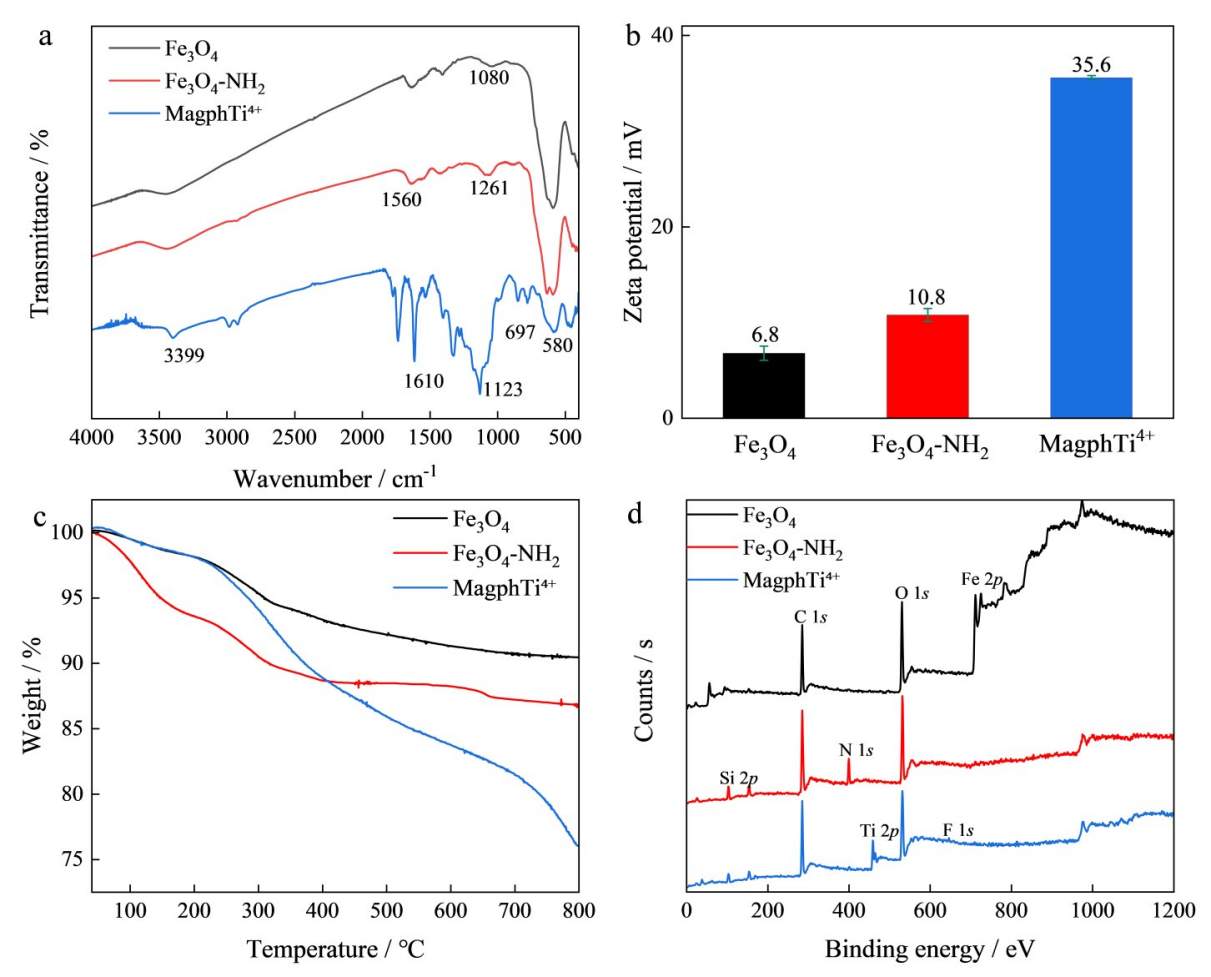
Fe_3_O_4_， Fe_3_O_4_-NH_2_和MagphTi^4+^的表征

对Fe_3_O_4_、Fe_3_O_4_-NH_2_、MagphTi^4+^进行了Zeta电位和DLS粒度分析。图3b展示了弱酸性条件下MagphTi^4+^的表面电荷，相较于Fe_3_O_4_和Fe_3_O_4_-NH_2_，MagphTi^4+^表面固定有大量Ti^4+^，在酸性条件下具有强正电荷，可与带负电的磷肽结合。DLS分析表明MagphTi^4+^在水中的尺寸约为270 nm。

图3c是对Fe_3_O_4_、Fe_3_O_4_-NH_2_、MagphTi^4+^的热重分析结果，相对Fe_3_O_4_和Fe_3_O_4_-NH_2_在完整加热过程中质量的轻微降低，MagphTi^4+^的重量损失大约为24.0%，在30~225 ℃范围内的轻微失重（约2.5%）是由于水和有机小分子的蒸发；在300~490 ℃范围内发生了明显的失重（大约21.5%），这是因为后修饰基团的分解导致的。以上结果说明MagphTi^4+^具有良好的热稳定性。

最后，采用X射线光电子能谱对Fe_3_O_4_、Fe_3_O_4_-NH_2_、MagphTi^4+^进行了表征。如图3d所示，与未经修饰的Fe_3_O_4_对比，经氨基化的磁性材料Fe_3_O_4_-NH_2_中出现N 1*s*（400 eV）的特征峰，说明成功修饰APTES。在MagphTi^4+^的图谱中，新出现了F 1*s*（689 eV）和Ti 2*p*（459 eV）两个峰，证明了2-三氟甲基-4-氨基苯甲酸和Ti^4+^的成功引入，间接表明了Ti^4+^和三氟甲基之间螯合作用的存在。此外，由SEM图像可知，硅层厚度（约为23 nm）高于XPS探测深度（<10 nm），因此，如图3d所示，Fe_3_O_4_-NH_2_与MagphTi^4+^的Fe 2*p* XPS信号几乎完全消失。

### 2.2 MagphTi^4+^富集磷肽的能力

以酶解后的10 pmol/μL *β*-酪蛋白为标准样品，考察MagphTi^4+^对磷肽段的富集能力。在不同溶剂条件下，MagphTi^4+^对目标肽段的吸附能力会发生变化。本研究通过控制单一变量法，分别考察不同的极性和酸度对缓冲溶液富集能力的影响。当缓冲溶液酸度为1% TFA时，优化不同体积分数（50%和70%）的ACN；当ACN体积分数为70%时，优化缓冲溶液酸度条件（1%和0.1% TFA）。结果表明，对未经富集的*β*-酪蛋白酶解液进行直接检测时几乎无法检测到磷肽的质谱峰（图4a）。当缓冲溶液为1% TFA的50%乙腈水溶液时，质谱图中存在杂峰且磷肽的峰信噪比相对较低（图4b）。缓冲溶液为含1% TFA的70%乙腈水溶液时，质谱图中几乎无杂峰且磷肽的峰信噪比相对较高（图4c）。当缓冲溶液为含0.1% TFA的70%乙腈水溶液时，质谱图中磷肽的峰信噪比相对较低（图4d）。因此，选取含1% TFA的70%乙腈水溶液作为后续富集实验中的缓冲溶液。

**图4:**
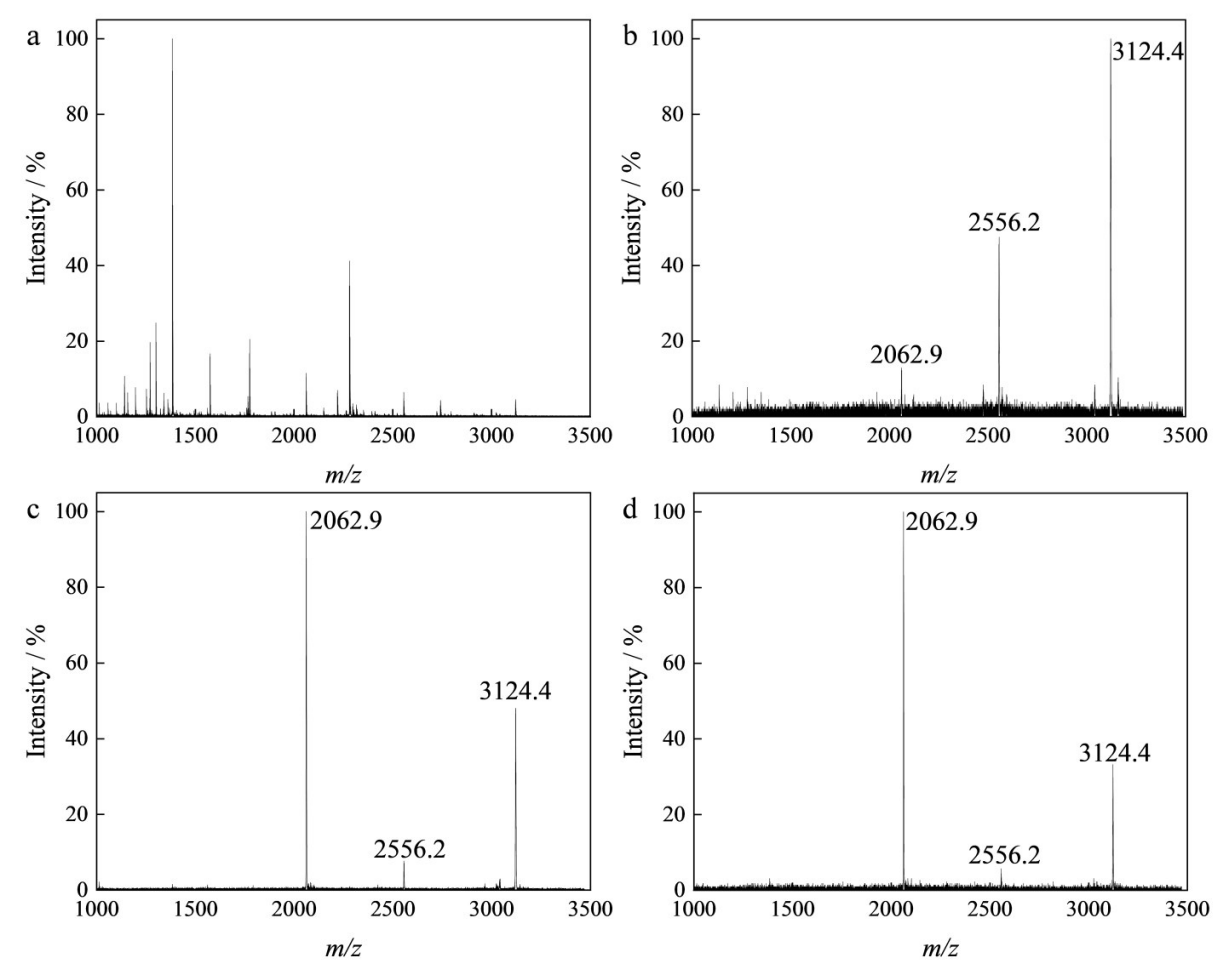
***β*-**酪蛋白酶解液的MALDI质谱图

在以上得到的最佳实验条件下考察方法的灵敏度，将酶解后*β*-酪蛋白溶液进行稀释（40、4和0.4 fmol/μL）。由图5可见，本方法可从0.4 fmol/μL的样品中检测到3条磷肽（*m/z* 2 062.9、2 556.2和3 124.4），且*S*/*N>*3，对比于近年其他文献所报道的方法（表1），本方法具有较高灵敏度，优于近年的大多数文献方法。

**图5:**
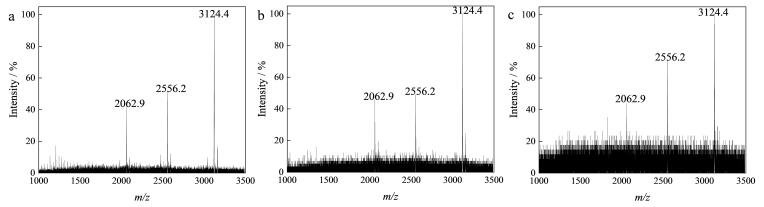
MagphTi^4+^富集后 ***β*-**酪蛋白酶解液的MALDI质谱图

**表 1:** 不同材料对磷肽富集后的分析结果

Material	LOD/（fmol/μL）	Selectivity （*β*-casein∶BSA， mass ratio）	Ref.
GO@PEI@CS@Ti^4+^	5	1∶100	［^38^］
SPIOs@PVP-PEI@MOF@Arg	10	1∶1000	［^39^］
Fe_3_O_4_@GO@PEI@Ti^4+^	2.5	1∶1000	［^40^］
Fe_3_O_4_@ILI-01@Ti^4+^	0.5	1∶1000	［^41^］
Fe_3_O_4_@UiO-66-NH_2_-Zr^4+^ HFs	1.0	1∶200	［^42^］
MagphTi⁴⁺	0.4	1∶5000	this work

BSA： bovine serum albumin.

为考察该方法的选择性，将*β*-酪蛋白与非磷蛋白BSA的混合酶解液按照1∶5 000的质量比混合作为混合样品。MALDI质谱检测结果表明，对复杂样品进行直接检测时无法检测到磷肽的质谱峰（图6a）；经MagphTi^4+^富集后，非磷肽的峰被显著抑制，可观察到较强的磷肽信号峰（*m/z* 2 062.9、2 556.2、3 124.4）（图6b）。最后，为考察MagphTi^4+^的可重复利用性，选用10 pmol/μL *β*-酪蛋白酶解液作为标准品，在相同条件下，用MagphTi^4+^对*β*-酪蛋白酶解液进行反复富集和洗脱，对第1次和第6次富集的结果进行分析，如图6c和6d所示，该材料在重复使用6次后所富集到的磷肽与第1次富集的结果几乎无差异，仍可高特异性富集磷肽。

**图6:**
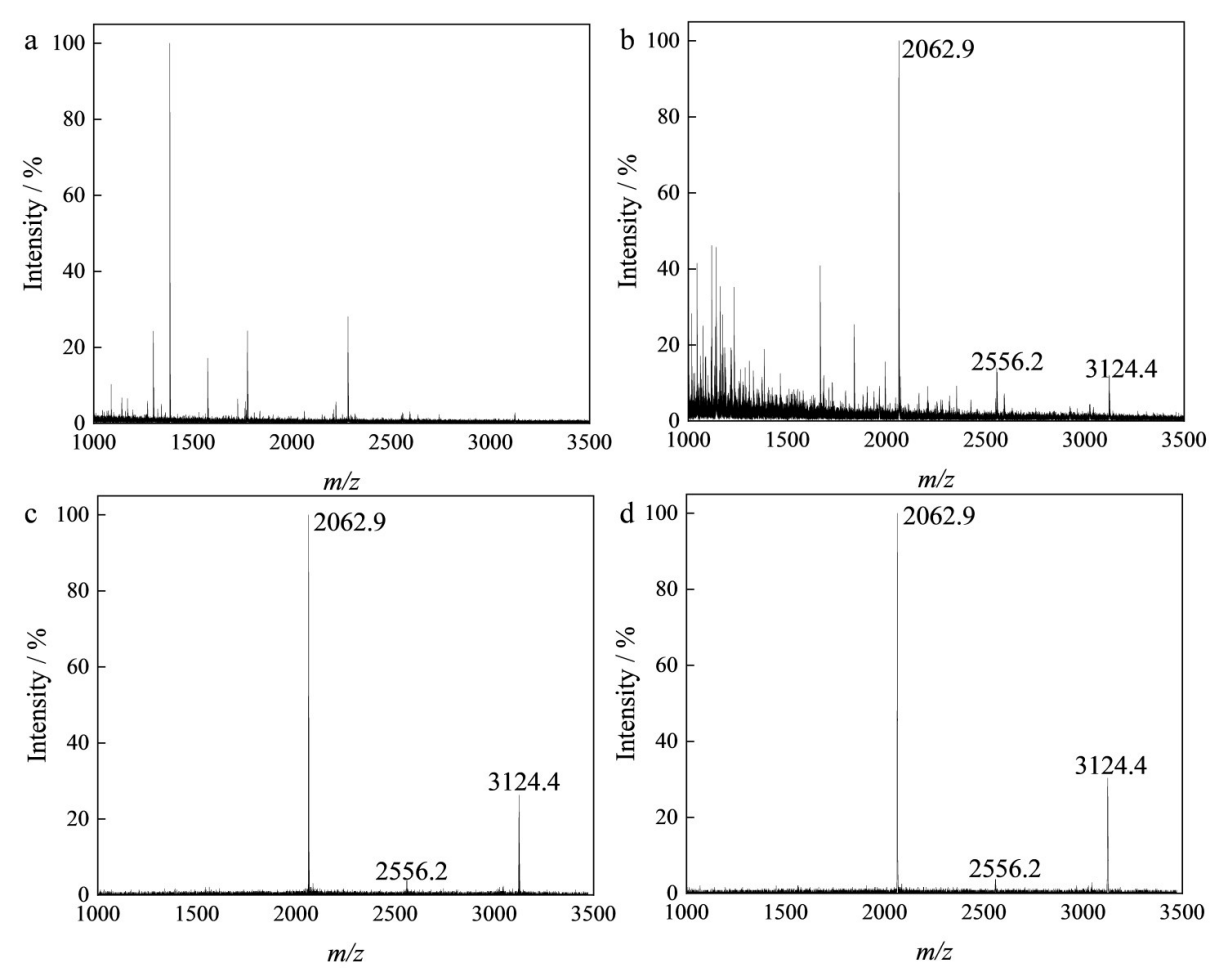
（a，b）混合样品和（c，d） ***β*-**酪蛋白酶解液的质谱图

### 2.3 实际样品分析

为了验证MagphTi^4+^在实际生物样品分析中对磷肽的富集能力，选择唾液和脱脂牛奶样品，在最优富集条件下对实际样品进行分析检测。如图7a所示，在未经富集的唾液样品中几乎检测不到磷肽。而经过MagphTi^4+^富集后，共检测到11条磷肽（图7b），其中包括4条多磷肽和7条单磷肽，表2为检测到的磷肽的详细信息。如图7c所示，在未经富集的脱脂牛奶样品中，没有检测到磷肽的信号，这是由牛奶样品中大量非磷肽的干扰造成的。相比较，经MagphTi^4+^富集后，在牛奶样品中检测到20条磷肽（图7d），其中包括9条多磷肽和11条单磷肽，表3为检测到的磷肽的详细信息。以上结果说明所制备的MagphTi^4+^具备应用于实际样品分析的能力。

**图7:**
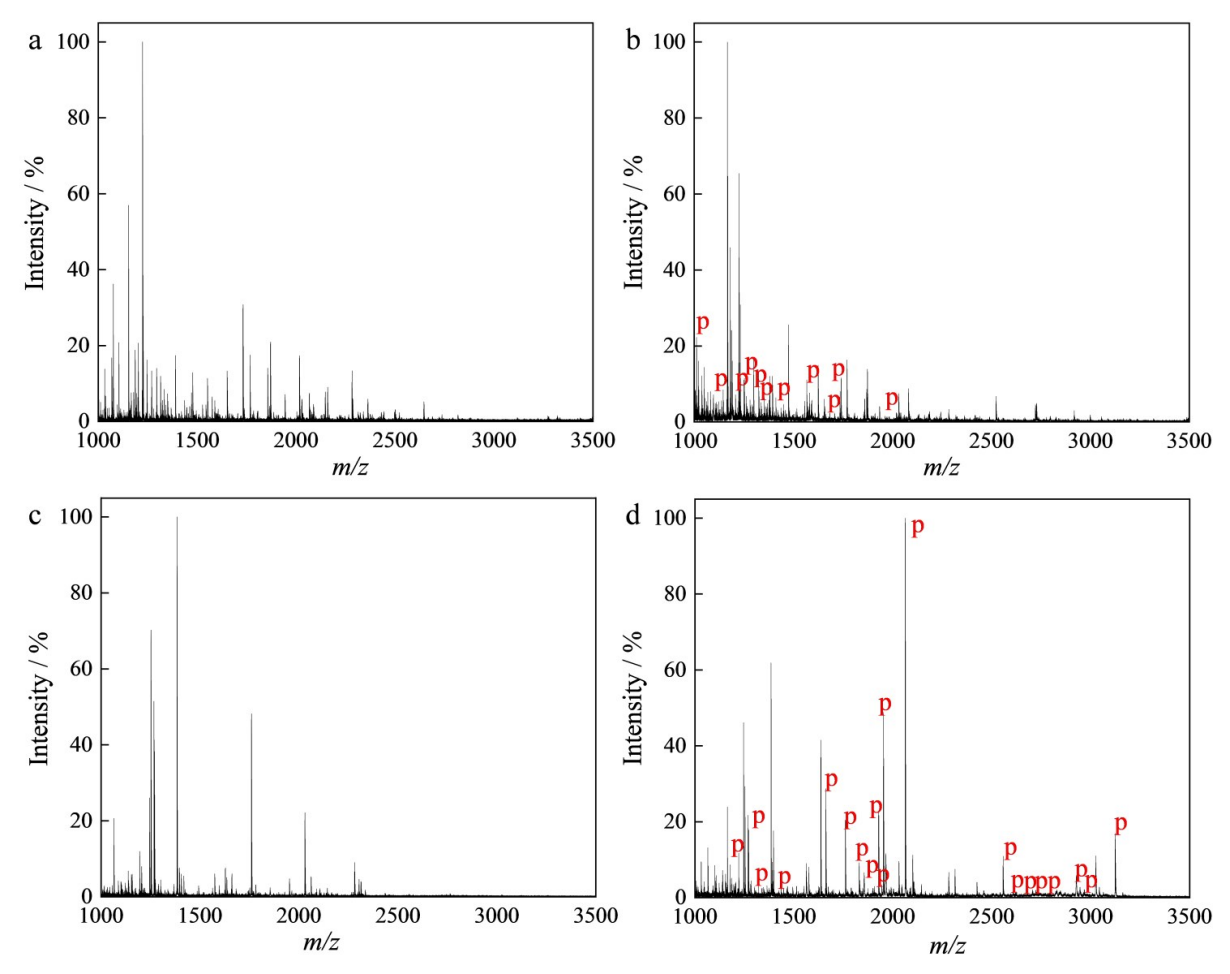
（a，b）唾液样品和（c，d）脱脂牛奶样品的质谱图

**表 2:** 经MagphTi^4+^富集后唾液样品中的磷肽

No.	*m/z*	Peptide sequence
1	998.06	ssEEKFL
2	1159.15	ssEEKFLR
3	1187.70	DsSEEKFLR
4	1231.64	SsEEKFLRR
5	1270.75	DssEEKFLR
6	1305.53	GPPPQGDKsRSP
7	1424.19	DssEEKFLRR
8	1589.70	GGDsEQFLDEERQ
9	1703.96	DGGDsEQFLDEERQ
10	1731.92	GPPAQGGsKSQSARAPPG
11	2003.99	VISDGGDsEQFIDEERQ

* All phosphopeptides obtained from each sample are numbered in ascending order of *m/z*.

**表 3:** 经MagphTi^4+^富集后脱脂牛奶样品中的磷肽

No.	*m/z*	Peptide sequence
*α*1	1237.50	TVDMEsTEVF
*α*2	1267.92	YLGYLEQLLR
*α*3	1337.70	HIQKEDVsER
*α*4	1466.86	TVDMEsTEVFIK
*α*5	1660.79	VPQLEIVPNsAEER
*α*6	1759.75	HQGLPQEVLNENLLR
*α*7	1832.73	YLGEYLIVPNsAEER
*α*8	1847.82	DIGSEsTEDQAMEDIK
*α*9	1927.56	DIGsEsTEDQAMEDIK
*α*10	1943.79	DIGsEsTEDQAMoEDIK
*α*11	1952.68	YKVPQLEIVPNsAEER
*α*12	2619.25	NTMEHVsssEESIIsQETYK
*α*13	2678.23	VNELsKDIGsEsTEDQAMEDIK
*α*14	2704.06	QMEAEsIsssEEIVPNsVEA
*α*15	2720.89	QMEAEsIsssEEIVPNPNsVE
*α*16	2935.24	EKVNELsKDIGsEsTEDQAMEDI
*β*1	2062.14	FQsEEQQQTEDELQDK
*β*2	2556.46	FQsEEQQQTEDELQDKIHPF
*β*3	2965.98	ELEELNVPGEIVEsLsssEESI
*β*4	3122.68	RELEELNVPGEIVEsLsssEESI

*α* represents *α-*casein. *β* represents *β-*casein.

最后，将MagphTi^4+^用于结直肠癌患者血清外泌体的提取及外泌体磷肽的富集分析。如图8a、8b所示，由SEM、TEM表征可见，图中存在直径约90 nm的囊泡为血清外泌体；如图9所示，十二烷基硫酸钠聚丙烯酰胺凝胶电泳分析结果表明提取的样品中存在血清外泌体的蛋白质标志物，结合电镜和电泳的表征可证明材料对外泌体的特异性提取^［43］^，但对外泌体浓度的定量有一定的局限性。将所提取的外泌体进行裂解及酶解操作后，对比经MagphTi^4+^富集前后磷肽的质谱结果。如图8c所示，在未经MagphTi^4+^富集的质谱图中，由于血清外泌体样品中大量非磷肽及其他无机盐的干扰，磷肽的信号丰度极低，几乎观测不到。经MagphTi^4+^富集后，可以检测到4条磷肽，归属于血清中的纤维蛋白原（图8d），富集到的磷肽的详细信息见表4。综上，MagphTi^4+^具有从复杂生物样品中精准识别和快速分离外泌体和富集外泌体磷肽的能力。

**图8:**
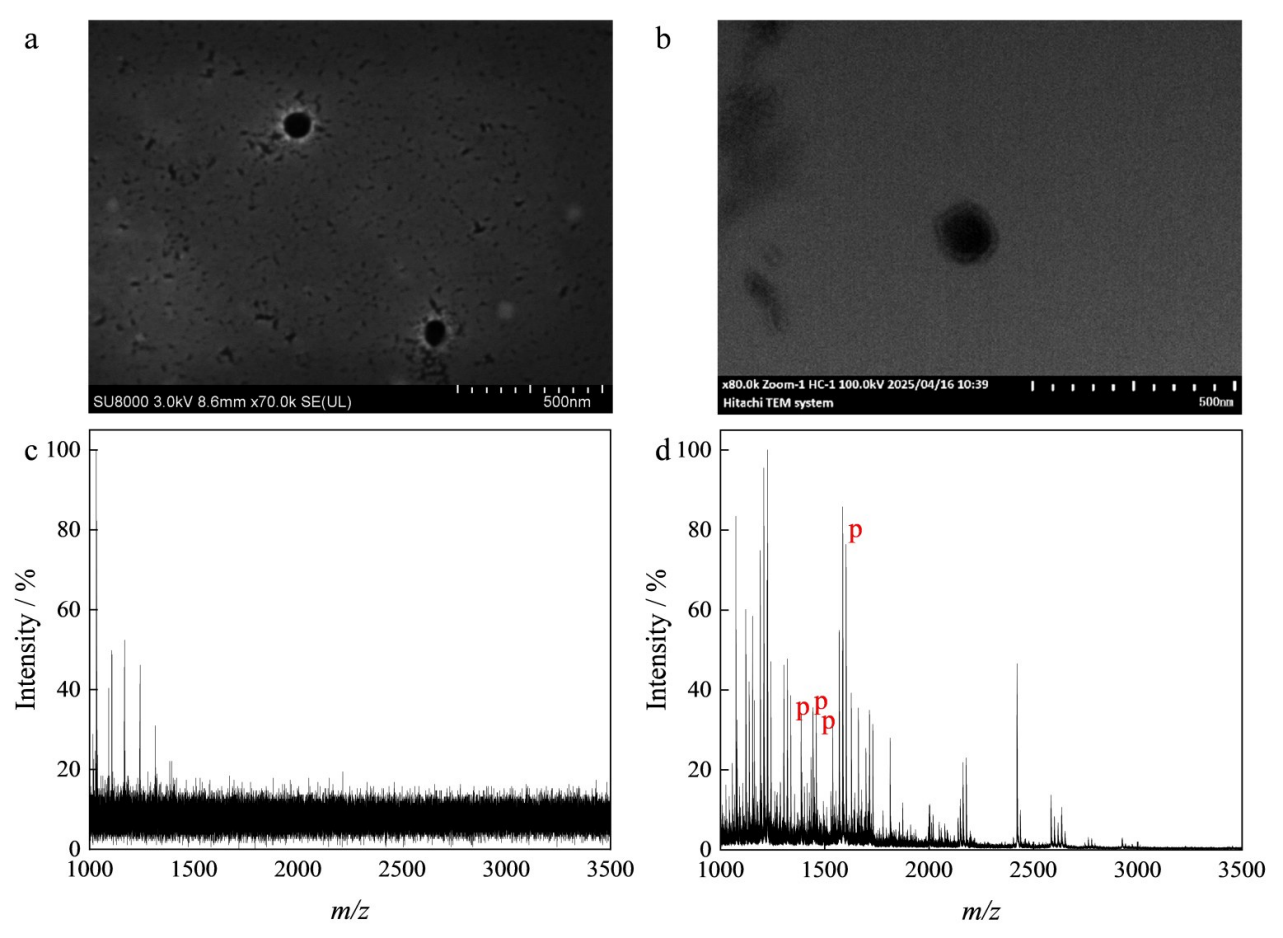
（a，b）对提取的外泌体的表征和（c，d）血清外泌体样品的质谱图

**图9:**
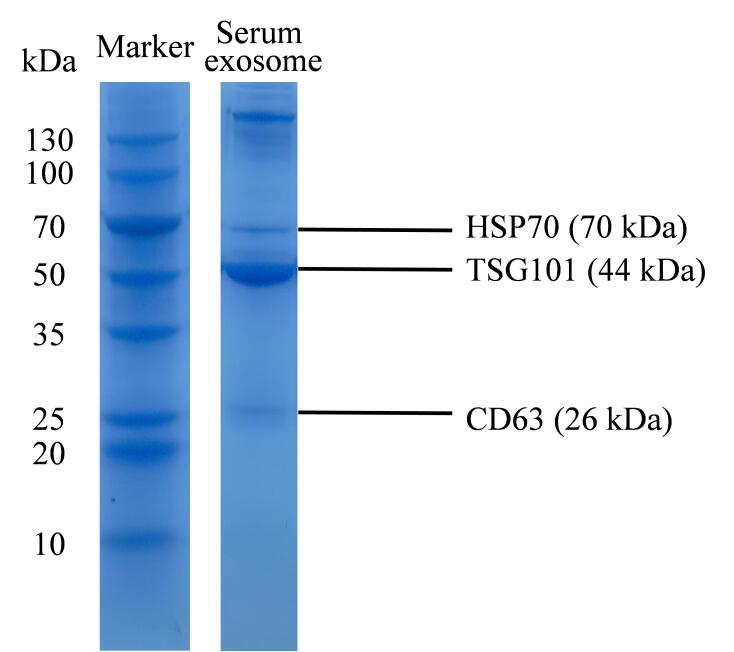
十二烷基硫酸钠聚丙烯酰胺凝胶电泳结果（考马斯亮蓝染色）

**表 4:** 经MagphTi^4+^富集后血清内外泌体中的磷肽

No.	*m/z*	Peptide sequence
1	1389.92	DsGEGDFLAEGGGV
2	1460.01	ADsGEGDFLAEGGGV
3	1545.06	DsGEGDFLAEGGGVR
4	1616.11	ADsGEGDFLAEGGGVR

## 3 结论

本研究制备了一种2-三氟甲基-4-氨基苯甲酸和Ti^4+^修饰的双功能磁性材料，能够对外泌体精准识别的同时实现对磷肽的高选择性分离富集。MagphTi^4+^材料以高磁响应性的优势实现了外泌体的快速分离，以Ti^4+^与磷肽间的IMAC机理实现了对磷肽的高效富集。结合MALDI-TOF MS检测手段，可实现对血清中外泌体磷肽的特异性检测。该方法克服了外泌体中低丰度磷肽检测的技术瓶颈，在临床样品磷酸化蛋白质组学分析中具有较高的潜力，为筛选高特异性肿瘤标志物提供了新思路。利用本研究开发的方法进行进一步研究有望为解析外泌体介导的磷酸化信号跨细胞传递机制、揭示肿瘤转移与免疫逃逸新靶点带来新的理解。
